# Brain cancer incidence rates and the presence of nuclear reactors in US states: a hypothesis-generating study

**DOI:** 10.1007/s10653-021-00896-0

**Published:** 2021-03-25

**Authors:** Mark R. Williamson, Marilyn G. Klug, Gary G. Schwartz

**Affiliations:** Department of Population Health, School of Medicine & Health Sciences, University of North Dakota, 1301 N Columbia Rd Stop 9037, Grand Forks, ND 58202-9037, USA

**Keywords:** Brain and CNS cancer, Cancer incidence, Epidemiology, Radiation, Nuclear reactors

## Abstract

**Background:**

The etiology of brain cancer is poorly understood. The only confirmed environmental risk factor is exposure to ionizing radiation. Because nuclear reactors emit ionizing radiation, we examined brain cancer incidence rates in the USA in relation to the presence of nuclear reactors per state.

**Methods:**

Data on brain cancer incidence rates per state for Whites by sex for three age groups (all ages, 50 and older, and under 50) were obtained from cancer registries. The location, number, and type of nuclear reactor, i.e., power or research reactor, was obtained from public sources. We examined the association between these variables using multivariate linear regression and ANOVA.

**Results:**

Brain cancer incidence rates were not associated with the number of nuclear power reactors. Conversely, incidence rates per state increased with the number of nuclear research reactors. This was significant for both sexes combined and for males in the ‘all ages’ category (β = 0.08, p = 0.0319 and β = 0.12, p = 0.0277, respectively), and for both sexes combined in the’50 and older’ category (β = 0.18, p = 0.0163). Brain cancer incidence rates for counties with research reactors were significantly higher than the corresponding rates for their states overall (p = 0.0140). These findings were not explicable by known confounders.

**Conclusions:**

Brain cancer incidence rates are positively associated with the number of nuclear research reactors per state. These findings merit further exploration and suggest new opportunities for research in brain cancer epidemiology.

## Introduction

Brain cancer and other nervous system cancers (henceforth “brain cancer”) accounted for approximately 23,890 new cases and 18,020 new deaths in the USA in 2020. The five-year relative survival for brain cancer is 35.8%. Brain cancers are extremely heterogenous, with more than one hundred classified types, each with potentially different risk factors (Louis et al. 2016). In children, most brain cancers are astrocytomas; in adults, most are glioblastomas (Ostrom et al. 2019).

The etiology of brain cancer is poorly understood (Barnholtz-Sloan et al. 2018). Incidence rates are higher among Whites than non-Whites and are higher among males than females. The only established environmental risk factor for brain cancer is exposure to ionizing radiation (Ostrom et al. 2014). A history of allergies and atopic diseases is associated with a modestly reduced risk (Wrensch et al. 2002). Higher socioeconomic status and higher educational attainment have been associated with increased risk in several studies (Cote et al. 2019; Khanolkar et al. 2015; Porter et al. 2015).

Brain cancer incidence rates within the USA show significant geographic variation. For example, among non-Hispanic White males, brain cancer incidence during 2013–2017 ranged from 7.3 per 100,000 in New Mexico (95% C.I. 6.2–8.4) to 9.6 per 100,000 in Idaho (95% C.I. 8.6–10.6). The reason(s) for this variation is unknown. The counties with the highest incidence of brain cancer in the USA are in southeastern Idaho, which is home to the Idaho National Laboratory, a facility that includes the largest number of nuclear reactors in the world (Cancer Data Registry of Idaho 2000; Strong, 2014). This suggested to us that the density of nuclear reactors might explain some of the geographic variation in brain cancer incidence rates. We therefore examined the distribution of brain cancer in US states in relation to the type and number of nuclear reactors per state.

## Methods

Five-year average incidence rates for brain cancer for the period of 2013–2017, stratified by sex and for both sexes together, were obtained from State Cancer Profiles (statecancerprofiles.cancer.gov). Rates were age-adjusted to the 2000 US Standard Population for non-Hispanic Whites in three age categories: all ages, 50 and older, and under 50. We selected non-Hispanic Whites because their population size and risk of brain cancer is greatest.

Data on nuclear power plant reactors were obtained from the US Nuclear Regulatory Commission (nrc.-gov/info-finder/reactors). Data on nuclear research and test reactors were obtained from the International Atomic Energy Agency Research Reactor Database (nucleus.iaea.org/RRDBAll). The active nuclear reactors (91 power plant reactors, 49 research and test reactors) were assigned to their state location. Because the average latency of radiation-induced gliomas is 10 years (Yamanaka & Hayano, 2015), we excluded reactors that began operation less than a decade before 2013, the start of our range of incidence rates.

There were three or fewer cases for females under age 50 in Hawaii; thus, data were not available for that stratum. Brain cancer data in state cancer profiles for White non-Hispanics also were not available for six states (DE, IL, KS, KY, MA, and PA). In order to estimate whether our analyses were influenced unduly by these missing values, we performed a sensitivity analysis in which we used data for Whites (including Hispanics) in place of the missing values for White non-Hispanics.

We excluded three reactors that fell outside of our time frame: a research reactor in California (ISSA Inherently Safe Subcrit); a power reactor in Alabama (Farley 1); and a power reactor in Tennessee (Watts Bar 2). A research reactor in New Mexico (White Sands Fast Burst Reactor) that was shut down in 2015 was included, in light of the brain cancer latency period noted above.

### Statistical analysis

For each age category, we used two-tailed t-tests for state-level brain cancer incidence rates, stratified by sex and for both sexes together, for each of the nuclear reactor categories. States with no reactors received a score of 0 and states with one or more nuclear reactors received a score of 1. This scoring was applied for power plant reactors, research reactors, and total reactors. We used ANOVA for state-level brain cancer incidence rates, both sexes together and stratified by sex, across four nuclear reactor status categories: no reactors, power reactors only, research reactors only, and both power and research reactors.

We used linear regression for state-level brain cancer incidence rates for each age and gender category vs. the number of nuclear reactors per state. Multivariate regressions were performed that included the number of power plant reactors and number of research reactors as predictor variables across the age and gender categories.

We considered socioeconomic status, education level, and radon levels as potential confounding variables and performed simple linear regressions on brain cancer incidence rates and each potential confounder. We used data on the median household income per state and percent of bachelor’s degrees per state from the American Community Survey for 2013–2017 to represent socioeconomic status and education levels, respectively (U.S. Census Bureau, 2017). We used the weighted mean residential radon level per state (in pCi/L) for radon levels, as described by Schwartz and Klug (2016). Radon data were not available for four states (AR, MN, NV, RI). None of the variables were associated with brain cancer incidence for any age or sex category at the p < 0.10 level. Consequently, these variables were not included in the final regression models.

Regressions were also analyzed using Poisson and log-normal distributions. Simple linear regression models were found to have the best fit (comparing Pearson Chi-square/df values) and therefore were used in the final analysis. Statistical analyses used SAS Studio V.3.8 (Cary, North Carolina, USA).

## Results

Mean state-wide brain cancer incidence rates for all ages were 7.2 (SD = 0.37) per 100,000 for both sexes, 8.5 (SD = 0.51) for males and 6.2 (SD = 0.35) for females. For persons 50 and older, incidence rates were 15.5 per 100,000 (SD = 0.71), for both sexes, 18.7 (SD = 1.24) for males and 12.7 (SD = 0.98) for females. For persons under 50, incidence rates were 4.1 per 100,000 (SD = 0.35) for both sexes, 4.6 (SD = 0.46) for males and 3.7 (SD = 0.36) for females. The average median household income was $57,848 (SD = $9,547), and the percent of the state’s population with bachelor’s degrees was 18.85% (SD = 2.82%). The mean weighted radon level was 3.07 pCi/L (SD = 1.52).

**Fig. 1:**
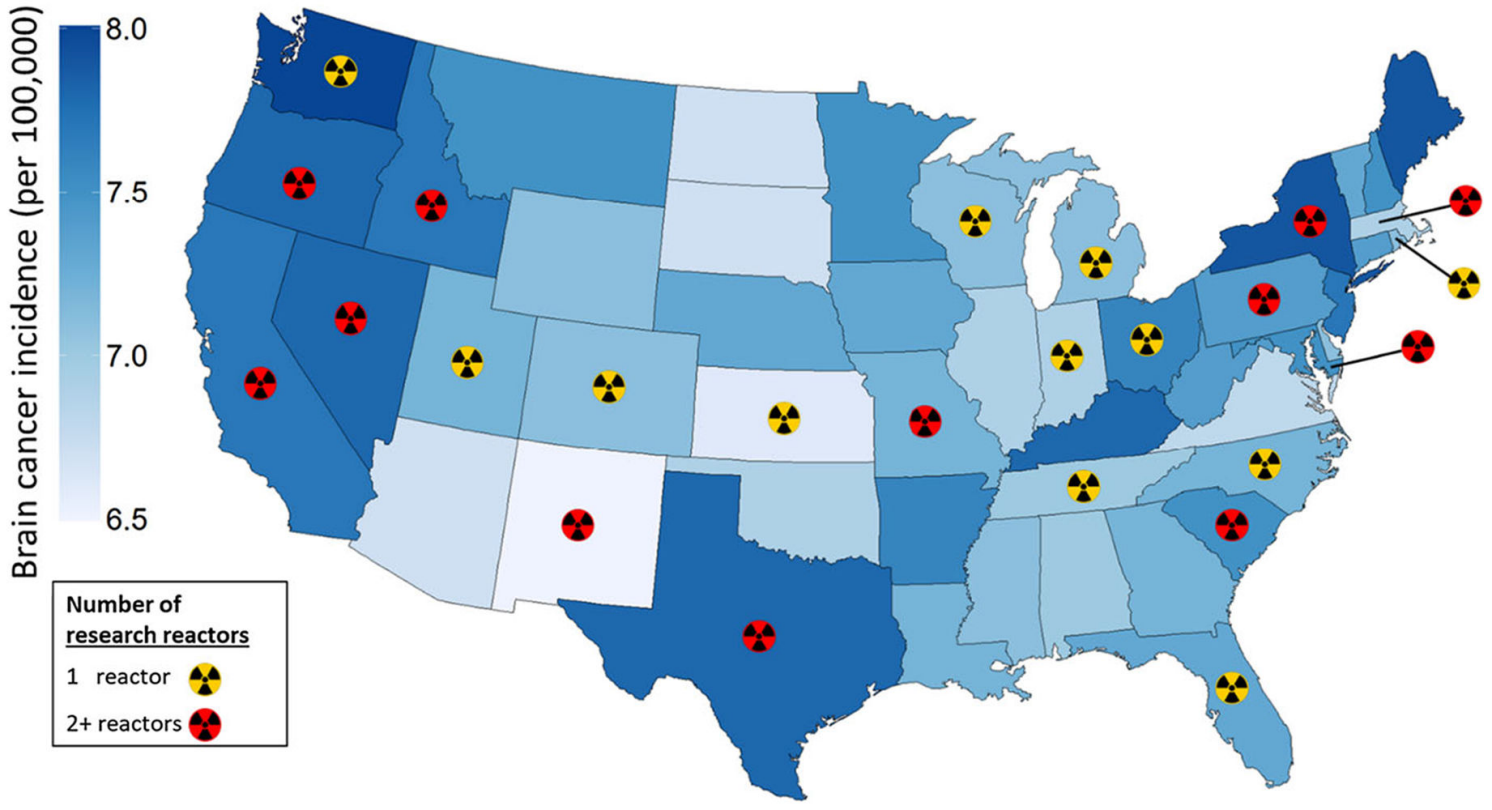
Map of brain cancer incidence rates and nuclear research reactor number. State-level map of brain cancer incidence rates per 100,000 for all ages, males and females combined. Rates are age-adjusted 5-year averages. Rates for 44 states are for Non-Hispanic Whites (NHWs). Six states (DE, IL, KS, KY, MA, and PA) did not have such rates available, and rates for Whites including Hispanics (WIH) were used. Alaska and Hawaii (not shown) have rates of 7.2 and 7.3, and both have 0 research reactors

Thirty-eight states had at least one nuclear reactor. Twenty-one states had no nuclear power reactors, twenty-six had no research reactors, and twelve had neither. The median number of reactors was 1, 0, 2, for power, research, and total (power and research) reactors. For research reactors, twenty-six states had no reactors, twelve had one, and twelve had two or more (Fig. 1). The state with the greatest number of total nuclear reactors [n = 11] was Pennsylvania.

Across the three age categories, incidence rates (per 100,000) were slightly but not significantly higher for states with vs. without nuclear reactors (for both sexes and total nuclear reactors; all ages: with = 7.29, without = 7.24; under 50: with = 4.134, without = 4.125; 50 and older: with = 15.55, without = 15.28). Similarly, there was no significant difference between the four categories of states (those with both reactors, power reactors only, research reactors only, none).

**Fig. 2:**
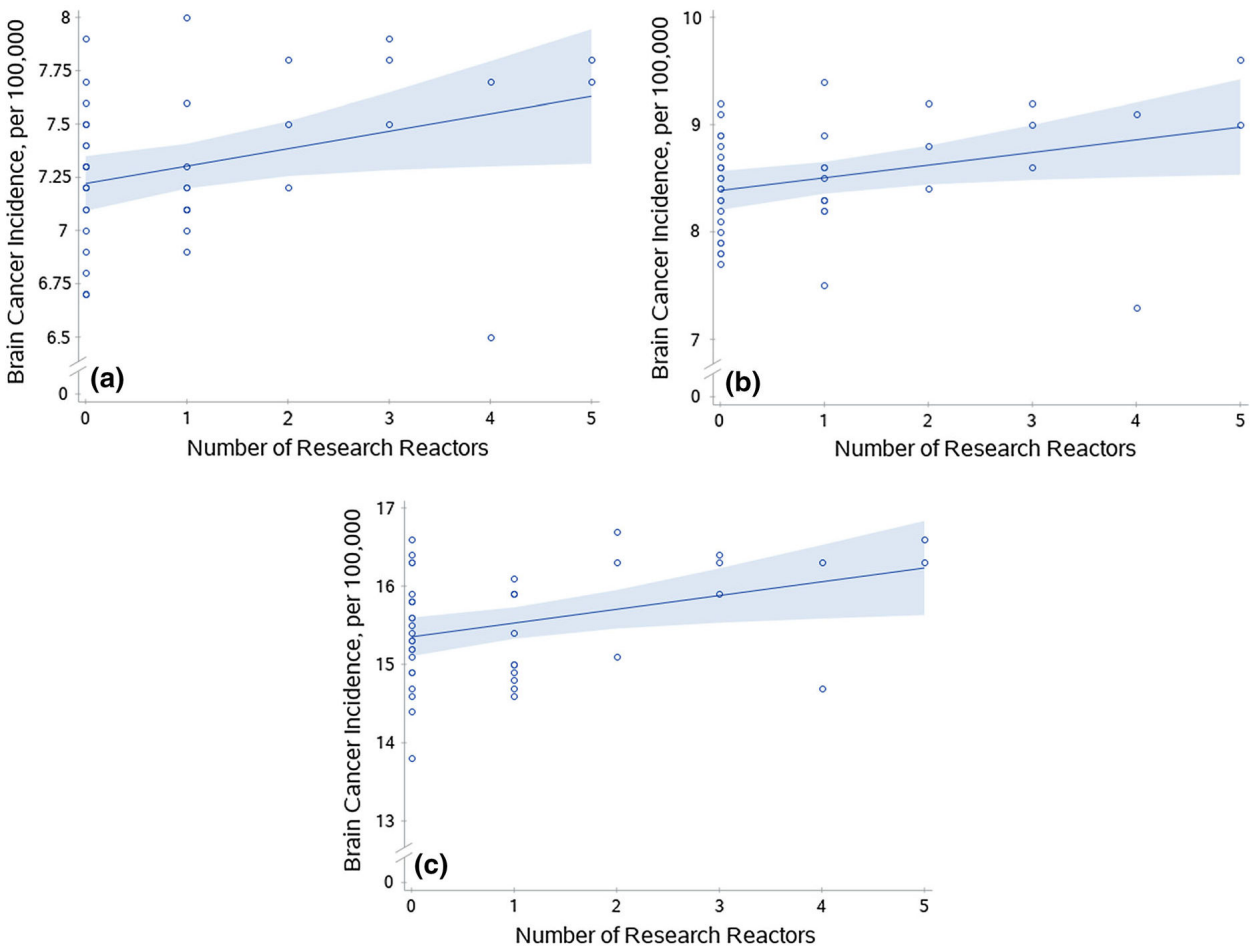
Brain cancer incidence rates and nuclear research reactors. Scatterplots of brain cancer incidence by state and the number of nuclear research reactors per state. **a**) Male and female combined incidence for all ages for research reactors (*p* = 0.0319). **b**) Male incidence for all ages for research reactors (*p* = 0.0277). **c**) Male and female combined incidence for ages 50 and older for research reactors (*p* = 0.0163). Lines were generated from prediction mean values from a simple linear model (research reactor/facility as only predictor variable) in SAS and bands are 95% confidence limits of the mean

Conversely, in regression models for the ‘all ages’ category, incidence rates were significantly related to the number of research reactors for both sexes combined (β = 0.08, t = 2.22, p = 0.0319) and for males alone (β = 0.12, t = 2.28, p = 0.0277) (Fig. 2a-b). This was also observed for the ‘50 years and older’ age category for both sexes combined (β = 0.18, t = 2.50, p = 0.0163) (Fig. 2c). Research reactors for males alone in the’50 years and older’ age category was of borderline significance (β = 0.25, t = 1.93, p = 0.0611). No models were significant for power reactor number. Furthermore, no model was significant for the ‘under 50’ category or for females alone (Table 1). All beta (β) coefficients in significant models were positive, indicating that brain cancer incidence rates increased per unit increase in reactor number.

Because six states did not report brain cancer incidence rates for Non-Hispanic Whites (NHW) separately, we performed a sensitivity analysis analyzing the data using incidence rates from the State Cancer Profiles for those states using Whites including Hispanics. The results differed little from the original analysis. Specifically, the associations for males and females combined and for males alone in the ‘all ages’ category remained significant (β = 0.08, t = 2.02, p = 0.0492; β = 0.011, t = 2.17, p = 0.0352), as did the associations for males and females combined for the’50 and older’ age category (β = 0.17, t = 2.40, p = 0.0203) for research reactors. The only material difference when using data for all fifty states was that the incidence rate by research reactor number for males alone for the’50 and older’ category became significant (β = 0.26, *t* = 2.07, *p* = 0.04037) (Supplemental Table 1).

We further explored the brain cancer incidence rates for states with research reactors at the county level. Brain cancer incidence rates were available for 30 of the 36 counties (83%) with research reactors. This included one county that also had a power reactor (Wake County, NC). Rates were suppressed in Bannock, Bonneville, Riley, Phelps, Anderson, and Whitman Counties. We created a “difference” variable by calculating the difference between the incidence rate for individuals 50 and older for the state from the corresponding incidence rates for counties with research reactors. After confirming the normality of the data, a two-sided, one-sample t-test was run on the difference variable. The mean rate for the state was 18.47 per 100,000 vs. 19.80 for 100,000 for the county. Thus, brain cancer incidence rates were significantly higher in counties with research reactors (t-value = 2.62, *p* = 0.0140). The mean difference, 1.50 (95% C.I. 0.33–2.67), indicates that, on average, counties with research reactors had approximately two more individuals per 100,000 diagnosed with brain cancer than the state in which they are embedded.

## Discussion

We tested the association of state-wide brain cancer incidence rates with the number and type of nuclear reactors. Across the three age categories, incidence rates did not differ significantly in binary comparisons comparing states with, vs. states without, nuclear reactors. However, in regression analyses when the number and type of nuclear reactors were analyzed, we found significant associations between brain cancer incidence rates and the number of research reactors. The regressions were significant for the age categories “all-ages” and “50 and older” but not at younger ages. These findings do not appear to be the result of confounding by suspected confounders as there were no appreciable correlations between brain cancer incidence rates and socioeconomic status, educational level and residential radon levels.

Several authors, (Cote et al. 2019; Khanolkar et al. 2015; Porter et al. 2015), but not all (Nilsson et al. 2018), reported positive associations between socioeconomic status and educational level and brain cancer. These variables were positively associated with brain cancer in our data but were not statistically significant. Similarly, in ecologic studies of brain cancer and residential radon levels, both positive (Ruano-Ravina et al. 2017) and null results (Berlivet et al. 2020) were reported at the municipality level in Spain and in France (respectively). A significant association between radon and brain cancer incidence was reported for a Danish cohort that used radon levels that were estimated based on housing and geologic characteristics (Brauner et al. 2013). Our null findings for brain cancer and radon in US states are consistent with null findings reported at the county level for several individual US states (Monastero & Meliker, 2020).

Numerous studies indicate that nuclear workers have an increased risk of brain cancer (e.g., Alexander 1991; Qu et al. 2018; Schubauer-Berigan et al. 2005)) although not all studies show this (Boice et al. 2006; Dupree-Ellis et al. 2000; Hunter et al. 2013; Kreuzer et al. 2015; Loomis & Wolf 1996; Lopez-Abente et al. 2001). It is unlikely that the size of the nuclear work force would be sufficient to substantially raise brain cancer incidence rates at the state level. Conversely, nuclear facilities could contribute to an increased risk of brain cancer via the emissions of radionuclides into the environment.

The issue of increased risks of cancer in the vicinity of nuclear facilities has been studied extensively (e.g., Boice et al. 2006; Lopez-Abente et al. 2001; Wing et al. 2011)). A comprehensive survey by the National Academy of Sciences (NAS) did not show increased mortality from cancer, including brain and nervous system cancers, for individuals living in US counties containing nuclear facilities vs. populations living in adjacent counties without such facilities (Jablon et al. 1991). Our (much cruder) comparison of brain cancer incidence rates at the state level also did not show an effect for large nuclear facilities. However, our analysis differs from the NAS approach in several respects. First, we posed a question not previously considered, concerning *the number of nuclear reactors*. We found that the incidence of brain cancer per state increased as the number of research reactors per state increased. Secondly, the NAS study focused on counties with nuclear power reactors, whereas our positive associations were found exclusively for counties with research reactors.

Power reactors are large facilities that use nuclear energy in order to generate electricity, whereas research reactors are smaller facilities that use nuclear reactions as a source of neutrons and radioisotopes for research. A potentially important difference between them is that research reactors typically use highly enriched uranium fuel that contains more uranium U-235 than the fuel used in power reactors (World Nuclear Association 2020).

Our findings could be due to chance, confounding, or a genuine relationship between the number of research reactors and brain cancer. Because we observed significant associations consistently for research reactors only, chance is an unlikely explanation. There was no evidence that the observed associations were due to the influence of known confounders, although the effects of unknown confounders cannot be excluded. For example, most research reactors are located on university campuses. It is possible that the associations between brain cancer incidence rates and research reactors could reflect better diagnosis in counties with universities. Alternately, the observed associations could reflect exposure to effluents from nuclear facilities.

Nuclear facilities release radioactive effluents both intentionally, as part of normal operations, and accidentally. These effluents contain known carcinogens. The effluent released by nuclear reactors in the greatest quantity is tritium (radioactive hydrogen, ^3^H), which is released as tritiated water. Tritiated water readily enters the water cycle and becomes ubiquitous in the environment (Calmon & Garnier-Laplace 2010). For example, tritium levels in residential wells near the Hanford nuclear site have been detected at 400 times the federal drinking water standard (EHS Today Staff 2000). Tritium can replace normal hydrogen in biochemical reactions and incorporates selectively in the brain, where it concentrates in nucleic acids (Etnier et al. 1984; Kowalska 1985; Van Bruwaene et al. 1982; US Nuclear Regulatory Commission 2019b; Major, 1980). Although tritium is a plausible cause of brain cancer, research reactors would be expected to generate less tritium than power reactors, which were not significantly associated with brain cancer. However, the manner in which these facilities release tritium conceivably could differ. Research power plants also release other radionuclides, e.g., nanoparticles of uranium, which accumulate in the brain and cause neurologic dysfunction (Petitot et al. 2013).

Our study has several limitations. First, different reactors may present different radiation risks and may have been active for different lengths of time. Secondly, we could not account for residential histories or exposure to other important sources of ionizing radiation (e.g., therapeutic radiation). Most importantly, these are ecologic (group level) data at the level of the state and the county. As such, they indicate only that brain cancer rates are higher in states and in counties with research reactors and not that individuals exposed to these reactors experienced increased risk.

Conversely, this study has several strengths. It is the first to test the a priori hypothesis that brain cancer incidence is associated with number of nuclear reactors and is the first to do so for research reactors. Our findings for the density of research reactors per state and brain cancer incidence rates are very consistent. If they are not causal, they invite speculation as to their true cause. Finally, the hypothesis that the association between number of nuclear research reactors and brain cancer incidence reflects exposure to reactor effluents is biologically plausible.

Our findings suggest areas for future research. For example, county level brain cancer incidence rates might be modeled as a function of nuclear facility density using techniques of geographic information systems (GIS). However, the statistical instability of incidence rates based on small case numbers in many counties may make that approach impractical (U.S. Nuclear Regulatory Commission 2019a). The association of brain cancer rates in older individuals with nuclear facilities makes predictions about secular trends in the most common brain cancer in adults, glioblastoma. It is notable that (unlike non-gliomas), the risk for glioblastoma in the USA increased markedly since the late 1970s (Li et al. 2018). Whether this increase is related to the proliferation of nuclear reactors in the USA (which began in the late 1950s) is an important question. Additionally, the potential role of radionuclides in brain cancer etiology could be tested via molecular epidemiology. Tritium and uranium can be measured in blood and urine (Belloni et al. 1983) as well as in solid tissues, including the brain (Hisamatsu et al. 1992; Ujeno et al. 1986). Thus, a case–control study of radionuclides in biological samples from individuals with and without brain cancer may be feasible.

In summary, incidence rates for brain cancer at the state level in the USA are significantly associated with the density of research reactors. This association may reflect the action of unknown confounders. Alternately, effluents from these facilities could underly this association.

## Supplementary Material

Suppl Material

## Figures and Tables

**Table 1:** Regression model results for brain cancer incidence and research reactor number. Multivariate regression model results that include predictor variables, beta (β) coefficients, t-values, and p-values. Results were significant only for research reactors alone, so a simple linear regression model followed each significant result for use in graph creation. Significant results are given in bold

Age Category	Sex	Predictor(s)	β-coefficient(s)	*t*-value(s)	*p*-value(s)
All ages	Males and Females	Power reactors	0.02	0.89	0.3785
		Research reactors	0.08	2.21	0.0330
All ages	Males and Females	Research reactors	0.08	2.22	0.0319
All ages	Males	Power reactors	0.03	0.88	0.3846
		Research reactors	0.12	2.27	0.0288
All ages	Males	Research reactors	0.12	2.28	0.0277
All ages	Females	Power reactors	0.03	0.97	0.3364
		Research reactors	0.05	1.38	0.1739
50 +	Males and Females	Power reactors	0.06	1.21	0.2327
		Research reactors	0.18	2.51	0.0163
50 +	Males and Females	Research reactors	0.18	2.50	0.0163
50 +	Males	Power reactors	0.13	1.31	0.1980
		Research reactors	0.25	1.93	0.0611
50 +	Females	Power reactors	0.04	0.48	0.6372
		Research reactors	0.08	0.75	0.4596
< 50	Males and Females	Power reactors	0.01	0.38	0.7062
		Research reactors	0.05	1.39	0.1733
< 50	Males	Power reactors	0.00	−0.10	0.9218
		Research reactors	0.07	1.48	0.1465
< 50	Females	Power reactors	0.02	0.75	0.4562
		Research reactors	0.03	0.83	0.4093

## Data Availability

Incidence data are available at https://statecancerprofiles.cancer.gov/data-topics/incidence.html; Nuclear reactor data are available at https://www.nrc.gov/info-finder.html
