# Supplementary material for: Brain cancer incidence rates and the presence of nuclear reactors in US states: a hypothesis-generating study
Source: Environ Geochem Health. 2021 Mar 25;43(10):3967–75. doi: 10.1007/s10653-021-00896-0 (PMC8463636; doi:10.1007/s10653-021-00896-0)
Supplement: Supplementary file 1 — Supplementary file1 (DOCX 13 kb) [file 10653_2021_896_MOESM1_ESM.docx]

**Supplemental Table 1. Sensitivity analysis.** Comparison of original analysis (Non-Hispanic white brain cancer incidence rates only) to re-analysis (inclusion of whites including Hispanic brain cancer incidence rates for the 6 states with missing NHW data). Significant results are shown in bold. Models for reactors with females alone or ages under 50 are not included as they were not significant for both analyses.

|  | | | Non-Hispanic whites (n=44) | | Non-Hispanic whites (n=44) and whites including Hispanics (n=6) | |
| --- | --- | --- | --- | --- | --- | --- |
| **Age Category** | **Sex** | **Predictor(s)** | **t-value(s)** | **p-value(s)** | **t-value(s)** | **p-value(s)** |
| All ages | Males and Females | Power reactors + Research reactors | 0.89  **2.21** | 0.3785  **0.0330** | 0.32  1.99 | 0.7537  0.0523 |
| All ages | Males and Females | Research reactors | **2.22** | **0.0319** | **2.02** | **0.0492** |
| All ages | Males | Power reactors + Research reactors | 0.88  **2.27** | 0.3846  **0.0288** | 0.66  **2.14** | 0.5149  **0.0378** |
| All ages | Males | Research reactors | **2.28** | **0.0277** | **2.17** | **0.0352** |
| 50+ | Males and Females | Power reactors + Research reactors | 1.21  **2.51** | 0.2327  **0.0163** | 0.64  **2.37** | 0.5525  **0.0219** |
| 50+ | Males and Females | Research reactors | **2.50** | **0.0163** | **2.40** | **0.0203** |
| 50+ | Males | Power reactors + Research reactors | 1.31  1.93 | 0.1980  0.0611 | 0.96  **2.05** | 0.3426  **0.0464** |
| 50+ | Males | Research reactors | 1.92 | 0.0616 | **2.07** | **0.0437** |
| All ages | Males and Females | Power facilities + research facilities | 1.27  **2.09** | 0.2098  **0.0425** | 0.55  1.60 | 0.5833  0.1163 |
| All ages | Males and Females | Research facilities | **2.07** | **0.0447** | 1.61 | 0.1141 |
